# Advances in CDK4 and 6 Inhibitors: Transforming Breast Cancer Treatment

**DOI:** 10.3390/cancers17050760

**Published:** 2025-02-24

**Authors:** Sonia Santander Ballestín, María Abadía Labena, Ana Avedillo-Salas, Cristina Marco Continente, Marina Arribas Blázquez, María José Luesma Bartolomé

**Affiliations:** 1 Department of Pharmacology, Phisiology, and Legal-Forensic Medicine, Faculty of Health and Sports Sciences, University of Zaragoza, 22001 Huesca, Spain; 2 Specialist in Anesthesiology and Reanimation Service, Universitary Clinic Hospital “Lozano Blesa”, Servicio Aragonés de Salud, 50009 Zaragoza, Spain; mabadia@salud.aragon.es; 3 Department of Pharmacology, Phisiology, and Legal-Forensic Medicine, Faculty of Medicine, University of Zaragoza, 50009 Zaragoza, Spain; anaavedillo@unizar.es; 4 General and Digestive Surgery, Mérida Hospital Complex, 06800 Mérida, Spain; 5 Department of Pharmacology and Toxicology, Veterinary Faculty, Universidad Complutense de Madrid, Avda/Puerta de Hierro s/n, 28040 Madrid, Spain; marrib01@ucm.es; 6 Department of Human Anatomy and Histology, Faculty of Medicine, University of Zaragoza, Domingo Miral s/n, 50009 Zaragoza, Spain; mjluesma@unizar.es

**Keywords:** breast cancer, metastasis, endocrine therapy, hormone therapy, cyclin-dependent kinase 4 and 6 inhibitors

## Abstract

Background and Objectives: Breast cancer is the most common malignant neoplasm worldwide and the most prevalent one among women. It represents the leading cause of cancer-related death among females. Cyclin-dependent kinase 4 and 6 inhibitors disrupt the cell cycle, inducing cellular senescence and, ultimately, apoptosis. Consequently, they have become a novel type of adjuvant therapy for the treatment of advanced or metastatic breast cancer characterised by positive hormone receptors and human epidermal growth factor receptor 2 (HER-2) negative. Methods: A systematic review was conducted, analysing the available literature on cyclin-dependent kinase 4 and 6 inhibitors published over the last five years. The aim was to evaluate the efficacy and safety of adding these drugs to the standard endocrine therapy for this pathology. Results: The combination of cyclin-dependent kinase 4 and 6 inhibitors with endocrine therapy was shown to improve progression-free survival, overall survival, and chemotherapy-free intervals in patients who received this combination therapy. Conclusions: The addition of CDK4/6 inhibitors to endocrine therapy in the treatment of advanced or metastatic breast cancer with positive hormone receptors and HER-2 negative significantly improved PFS, median survival, and chemotherapy-free intervals compared with the use of hormonal treatments alone or in combination with a placebo. Currently, CDK4/6 inhibitors are becoming established as a new standard treatment for this pathology, offering lower toxicity than chemotherapy. However, it is necessary to deeply investigate the mechanisms of treatment resistance and develop effective therapies to overcome them.

## Simple Summary

Breast cancer is the most common malignant neoplasm worldwide and the most prevalent one among women. Cyclin-dependent kinase 4 and 6 inhibitors have become a novel form of adjuvant therapy for the treatment of advanced or metastatic breast cancer characterised by positive hormone receptors and human epidermal growth factor receptor 2 (HER-2) negative. These inhibitors are considered a new standard treatment for this pathology, offering lower toxicity than chemotherapy.

## 1. Introduction

### 1.1. Breast Cancer

Breast cancer is the most common type of cancer globally, with more than 2.2 million cases reported in 2023, and the most frequent malignancy among women, making it the leading cause of cancer-related death in females (5.8% of all cancer deaths; 14.6% of cancer-related deaths in women). Moreover, 1 in every 12 women will develop this type of tumour during her lifetime [1,2,3,4].

Breast cancer typically occurs between the ages of 35 and 80, with the highest incidence observed in the 45–65 age group, coinciding with hormonal changes associated with peri- and postmenopause. The incidence rate increases with age, which explains why the number of breast cancer cases has risen in recent years, as life expectancy has also increased [4].

This type of tumour is categorised into four distinct types based on the molecular markers expressed by neoplastic cells, as outlined in Table 1 [5].

The Luminal A and Luminal B subtypes are characterised by positive hormone receptor expression. The Luminal A subtype shows estrogen and progesterone receptor expression greater than 20%, no HER-2 expression, and a low proliferation index (Ki67 < 20%). The Luminal B subtype can be further divided into two subgroups: those expressing HER-2 and those which do not. Both subgroups exhibit estrogen receptor positivity, progesterone receptor expression ≤ 20%, and a high proliferation index (Ki67 ≥ 20%).

The HER-2 subtype lacks any type of hormonal receptor but expresses HER-2.

Finally, the basal-like or triple-negative subtype does not express hormonal receptors or HER-2.

### 1.2. Therapeutic Options

Currently, various therapeutic options are available for breast cancer, as summarised in Table 2. Breast cancer treatment must be individualised and is based on three main approaches: surgery, radiotherapy, and medical treatment (or systemic therapy).

Breast cancer surgery should aim to be breast-conserving (lumpectomy) whenever possible, provided that the tumour can be excised with clear margins. This approach yields the best cosmetic outcomes. However, when mastectomy is indicated, patients should be offered the option of breast reconstruction [6].

Radiotherapy is recommended after all lumpectomies and for mastectomies involving tumours larger than 5 cm, as well as cases of local extension, regardless of nodal status, or when there are four or more affected axillary nodes [6].

Systemic therapy includes chemotherapy, hormone therapy, immunotherapy, and targeted therapies.

Chemotherapy is determined based on the tumour stage, prognostic factors, tumour molecular subtype, as well as the patient’s age and comorbidities. Chemotherapy regimens which have demonstrated the greatest survival benefit include anthracycline- and taxane-based protocols. Chemotherapy is recommended to begin no later than six weeks after surgery [6].

Aside from that, hormone therapy is indicated for any patient with invasive breast cancer exhibiting hormone receptor positivity above 10%. For premenopausal women, treatment should always involve tamoxifen (a selective estrogen receptor modulator), whereas postmenopausal women may be treated with aromatase inhibitors (letrozole, anastrozole, or exemestane) or tamoxifen. Hormone therapy should be administered after chemotherapy rather than concurrently with it [6].

Also, targeted therapy is offered to patients whose tumours overexpress HER-2. Treatment involves trastuzumab, a humanised monoclonal antibody which selectively blocks the HER-2 receptor, thereby inhibiting neoplastic cell proliferation. This therapy is administered alongside chemotherapy [6].

Lastly, cyclin-dependent kinase 4 and 6 inhibitors are included within the group of targeted therapies. This review focuses on this specific class of drugs.

### 1.3. Cyclin-Dependent Kinase 4 and 6 Inhibitors

Cyclin-dependent kinases are proteins involved in tumour cell growth and are implicated in resistance to hormone therapy. Their application has been proven to be beneficial in various types of cancer, including breast cancer, soft tissue sarcomas, and non-small-cell lung cancer.

The role of CDK4 is that of a prognostic biomarker in soft tissue sarcomas and the synergistic effect of its inhibition in dedifferentiated liposarcoma sequential treatment [7].

Numerous molecular studies suggest that the CDK4 and 6 pathway may be hyperactivated in breast cancers with positive hormone receptors. Preclinical trials have demonstrated that these drugs act synergistically with endocrine therapy in the treatment of metastatic breast cancer and may even serve as a therapeutic alternative for tumours resistant to endocrine therapy. However, the first CDK inhibitors tested in various studies exhibited unacceptable toxicity profiles (leading to treatment discontinuation) and limited clinical benefit [8,9].

Subsequently, in 2012, Pfizer introduced palbociclib, a highly selective inhibitor of cyclin-dependent kinases 4 and 6. The PALOMA-1 study (a randomised, phase II clinical trial) confirmed its safety and clinical benefit, leading to its approval by the Food and Drug Administration (FDA) [9].

Currently, the drugs used to block this pathway are ribociclib, palbociclib, and abemaciclib, which are typically administered in combination with hormone therapy [5].

**Figure 1:**
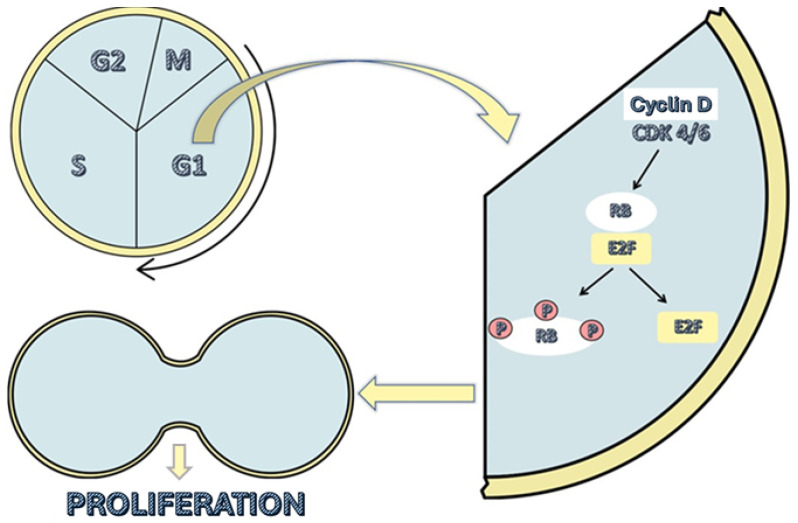
Cell cycle phases and the action of cyclin-dependent kinases 4 and 6. CDK4/6 = cyclin-dependent kinases 4 and 6; RB = retinoblastoma protein; E2F = transcription factor for DNA replication; P = phosphate.

These drugs are administered orally, and their function is to inhibit cyclin-dependent kinases 4 and 6 (CDK4 and CDK6, respectively). These kinases promote the transition from the G1 phase of the cell cycle to the S phase by causing the release of the E2F transcription factor. The transcriptional function of E2F is inhibited by the retinoblastoma suppressor protein (RB), as it directly binds to the activation domain of E2F when it is not phosphorylated, thereby blocking it. At the beginning of the G1 phase, RB is dephosphorylated and thus blocks the activation of E2F. At this point in the cell cycle, the presence of growth factors increases the levels of cyclin D, which bind to CDK4 and 6, forming a complex which combines with a third protein (p21 or p27) and resulting in an enzyme that phosphorylates the RB protein. This phosphorylation releases the E2F transcription factor, allowing it to function (Figure 1) [8].

By inhibiting CDK4/6, these drugs prevent RB phosphorylation and E2F release, halting the cell’s progression from the G1 phase to the S phase of the cell cycle. This ultimately results in cellular senescence and apoptosis [10,11,12].

## 2. Justification and Aims

The potential to establish cyclin-dependent kinases 4 and 6 (CDK4/6) as a novel therapeutic target, in conjunction with hormone therapy, for advanced or metastatic breast cancer characterised by positive hormone receptors and HER-2 negative could result in lower toxicity compared with conventional chemotherapy. This is because these drugs selectively inhibit the hyperactivated pathway in this tumour type. Given the high incidence, morbidity, and mortality rates associated with this condition, there is a clear need to conduct a systematic review of the available scientific evidence.

The primary aim of this article is to assess the efficacy and safety of this pharmacological group for the aforementioned therapeutic indication.

## 3. Materials and Methods

This systematic review was conducted by searching the PubMed, Cochrane, and Web of Science databases using the following algorithm (based on MeSH terms):

“((((ALL=(breast neoplasm)) AND ALL=(neoplasm metastasis)) AND ALL=(cyclin-dependent kinase inhibitor proteins)) OR ALL=(cyclin-dependent kinase 6)) OR ALL=(cyclin-dependent kinase 4)”

The inclusion criteria were as follows:

- Articles available in full text and free of charge;
- Results published from 2017 to 2024 (last 7 years);
- Clinical trials (randomised or non-randomised);
- Studies involving treatments with cyclin-dependent kinase 4 and 6 inhibitors (palbociclib, ribociclib, abemaciclib, or dalpiciclib);
- Studies which used these drugs in humans.

The remaining inclusion criteria, following the PICO strategy, are summarised in Table 3.

Exclusion criteria were defined as follows:

- Any other type of study;
- Application of cyclin-dependent kinase 4 and 6 inhibitors to other types of neoplasms;
- Studies involving patients with breast cancer with negative hormone receptors or HER-2 positive;
- Studies involving patients with early-stage breast cancer.

During this process, a total of 2029 articles were screened, of which 1806 were excluded at the initial stage after evaluating the titles and abstracts because they did not address advanced or metastatic breast cancer with positive hormone receptors and HER-2 negative.

After reviewing 223 articles in full, 171 were excluded for the following reasons:

- They did not compare one treatment with another treatment or placebo;
- They were not available in full text and free of charge;
- They were written in a language other than English or Spanish;
- They did not analyse PFS or OS;
- They analysed data from two different clinical trials together.

Of the 194 preselected articles, 23 were ultimately included in the review.

**Figure 2:**
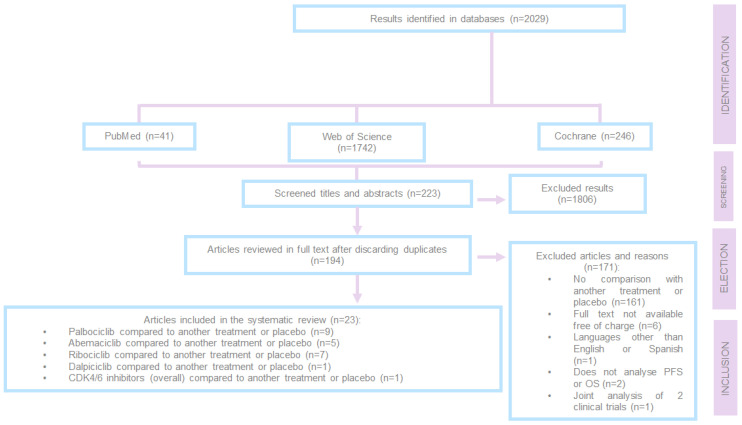
PRISMA 2009 flow chart. PFS = progression-free survival; OS = overall survival; n = total number.

This study was conducted according to the Preferred Reporting Items for Systematic Reviews and Meta-Analyses (PRISMA) statement [13,14]. The PRISMA flowchart (Figure 2) summarises the search process. The protocol has been registered in the PROSPERO database (registration number: 636389).

For the analysis of the clinical trials, we extracted and analysed the data according to the following parameters: progression-free survival (PFS), global survival (OS), and percentage of patients who suffered adverse effects.

## 4. Results

The efficacy and safety results are shown for each treatment.

### 4.1. Efficacy

#### 4.1.1. Ribociclib

Ribociclib is one of the cyclin-dependent kinase 4 and 6 inhibitors (whose molecular formula is C₂^3^H₃₀N₈O₃) approved by the FDA for the treatment of metastatic or advanced breast cancer with positive hormone receptors and HER-2 negative.

The MONALEESA-7 study is a randomised clinical trial (1:1) which included pre- or perimenopausal patients who received ribociclib or a placebo alongside goserelin and a non-steroidal aromatase inhibitor (NSAI) or tamoxifen. Participants were followed for a median of 53.4 months (cut-off date: 29 June 2020). In this study, patients were divided into two age groups: <40 years and ≥40 years. In patients <40 years in the ribociclib treatment group, the median overall survival (OS) was 51.3 months, compared with 40.5 months in the placebo group (HR: 0.65; 95% CI [0.43–0.98]), representing a 10.8-month increase. In patients ≥40 years old, the median OS was 58.8 months in the ribociclib group, compared with 51.7 months in the placebo group, though this difference was not statistically significant (HR: 0.81; 95% CI [0.62–1.07]) [15,16].

Moreover, the MONALEESA-2 study was a phase III, randomised clinical trial (1:1) which included 668 postmenopausal women with advanced breast cancer, positive hormone receptors, and HER-2 negative. They received first-line treatment with ribociclib and letrozole or letrozole combined with a placebo. At the cut-off date (10 June 2021), the median follow-up was 79.9 months. By this time, 400 deaths had occurred: 54.2% (181 patients) in the ribociclib group and 65.6% (219 patients) in the placebo group. At this stage, the combination of ribociclib and letrozole showed an increase in the median OS compared with the placebo group (63.9 months versus 51.4 months, respectively; HR: 0.76, 95% CI [0.63–0.93]; *p* = 0.04), resulting in a 12.5-month increase in the median OS. Additionally, at 6 years of treatment, the median survival rate was 44.2% for the ribociclib group compared with 32.0% for the placebo group. The combination treatment also delayed the need for chemotherapy (chemotherapy-free survival), with the patients receiving ribociclib showing a median of 50.6 months versus 38.9 months in the placebo group (HR: 0.74; 95% CI [0.61–0.91]), gaining an average of 11.7 months of chemotherapy-free survival [17,18].

Finally, the MONALEESA-3 study was a phase III, randomised (2:1), placebo-controlled clinical trial which included postmenopausal patients with advanced breast cancer, positive hormone receptors, and HER-2 negative. This study investigated the combination of ribociclib and fulvestrant in both first- and second-line treatments. The median follow-up was 39.4 months (cut-off date: 3 June 2019). The combination of ribociclib and fulvestrant demonstrated an increase in the median overall survival compared with the fulvestrant and placebo group (median OS in the ribociclib group not achieved versus 40 months in the placebo group; HR: 0.724, 95% CI [0.568–0.924]; *p* = 0.00455). This benefit was consistent across all subgroups, including those receiving the treatment in the first line (median OS in the ribociclib group not achieved versus 45.1 months in the placebo group; HR: 0.700; 95% CI [0.530–1.004]), with no significant difference in this latter subgroup. On the other hand, the median progression-free survival (PFS) in the subgroup treated with ribociclib and fulvestrant in the first line was 33.6 months, compared with 19.2 months in the placebo group (HR: 0.546; 95% CI [0.415–0.718]). Patients treated with the combination of ribociclib and fulvestrant took longer, on average, to require chemotherapy or die compared with those treated with fulvestrant and a placebo (39.8 versus 29.4 months, respectively; HR: 0.670; 95% CI [0.542–0.830]) [19].

#### 4.1.2. Palbociclib

Palbociclib is another drug belonging to the class of selective cyclin-dependent kinase 4 and 6 inhibitors, whose molecular formula is C₂₄H₂₉N₇O₂.

Its activity was studied in PALOMA-1, a phase II, randomised (1:1), open-label clinical trial which included 165 postmenopausal women with advanced breast cancer, positive hormone receptors, and HER-2 negative (18). One arm of the study received palbociclib (125 mg/24 h) and letrozole (2.5 mg/24 h), while the other received only letrozole (same dosage). The median follow-up time was 64.7 months (cut-off date: 30 December 2016). In the group treated with palbociclib combined with endocrine therapy, the median OS was 37.5 months (95% CI [31.4–47.8]) compared with 34.5 months in the letrozole-only group (95% CI [27.4–42.6]) (HR: 0.897; 95% CI [0.623–1.294]; *p* = 0.281), although this difference was not statistically significant [20].

Another study, PALOMA-2, was a phase III, double-blind, randomised (2:1) clinical trial which included 666 postmenopausal women with advanced breast cancer, positive estrogen receptors, and HER-2 negative who had not received any prior treatment. One arm (444 women) received treatment with palbociclib (125 mg/24 h, with a one-week break every 3 weeks of treatment) and letrozole (2.5 mg/24 h), while the other arm (222 patients) received endocrine therapy with a placebo. By the cut-off date (31 May 2017), the median follow-up was 37.6 months in the combined treatment group and 37.3 months in the placebo group. The combination of abemaciclib and letrozole showed an increase in PFS compared with the placebo arm, with a median of 27.6 versus 14.5 months, respectively (HR: 0.563; 95% CI [0.461–0.687]; *p* < 0.0001). Similarly, the group which received the therapy combining abemaciclib and letrozole took longer to require another systemic therapy (38.8 months (95% CI [34.4–not achieved]) compared with 28.8 months (95% CI [25.7–33.5])) in the group treated with letrozole and a placebo [21,22].

Another study which evaluated the addition of palbociclib to endocrine therapy was PALOMA-3, a phase III, randomised (2:1), double-blind, placebo-controlled clinical trial which included 521 patients (regardless of their menopausal status) with advanced breast cancer, positive hormone receptors, and HER-2 negative. One arm received treatment with palbociclib (125 mg/24 h with one week off for every 21 days of treatment) and fulvestrant (500 mg), while the other arm received fulvestrant (same dose) combined with a placebo. They were followed for a median of 44.8 months (cut-off date: 13 April 2018). By this time, the overall survival (OS) was 34.9 months (95% CI [28.8–40.0]) in the group receiving palbociclib and fulvestrant, compared with 28 months (95% CI [23.6–34.6]) in the placebo group. The 3-year median survival rate was 50% (95% CI [44–55]) in the group treated with palbociclib and fulvestrant, compared with 41% (95% CI [33–48]) in the placebo arm. It is worth noting that 311 patients had visceral metastasis, for which the OS was 27.6 months (95% CI [24.4–31.2]) in the palbociclib and fulvestrant arm, compared with 24.7 months (95% CI [20.8–31.8]) in the placebo group. On the other hand, 210 patients did not have visceral metastatic disease, and in this group, the OS was 46.9 months (95% CI [39.3–not achieved]) for those treated with the combination therapy, compared with 35.4 months (95% CI [24.6–not achieved]) for those treated with a placebo and fulvestrant [23].

Along the same line, PALOMA-4 was a phase III, double-blind, randomised (1:1) clinical trial which included postmenopausal Asian women who had not received prior systemic treatment and presented with advanced breast cancer, positive hormone receptors, and HER-2 negative. The patients (total number: 340) received palbociclib (125 mg/24 h orally, with 1 week off for every 3 weeks of treatment) and letrozole (2.5 mg/24 h orally without interruption) or a placebo combined with letrozole. They were followed for a median of 52.8 months (cut-off date: 31 August 2020). In this study, the median progression-free survival (PFS) was 21.5 months for the group which received palbociclib and letrozole, compared with 13.9 months in the placebo group (HR: 0.68; 95% CI [0.53–0.87]; *p* = 0.0012) [24].

Another study evaluating the addition of palbociclib to endocrine therapy was RENATA, a prospective study which included 128 Argentine women with breast cancer, positive hormone receptors, and HER-2 negative, 20% of which were premenopausal, while 44% had visceral metastasis. The objective of this study was to analyse the use of palbociclib combined with endocrine therapy in the real-world population. In most patients (63.9%), the CDK4/6 inhibitor was combined with aromatase inhibitors, while it was combined with fulvestrant in the remaining patients. The median PFS was 29.6 months (95% CI [19.5–38.8]) when used as first-line treatment and 24.2 months (95% CI [12.0–32.7]) when used as second-line or later therapy. Furthermore, the PFS was greater when palbociclib was combined with fulvestrant (32.7 months; 95% CI [9.3–33.4]) compared with when it was combined with aromatase inhibitors (29.6 months; 95% CI [18.1–43.6]). After 36 months of follow-ups, 7.2% of the patients who received the first-line treatment had died, whereas 26% of those who received the second-line treatment had died. The OS after treatment with palbociclib was 15.6 months (95% CI [4.8–26.3]) [25].

The final study to be analysed regarding the addition of palbociclib to standard hormone therapy for breast cancer was the one conducted by Orlandi et al., who carried out a retrospective study which included 74 women with metastatic breast cancer, 48 of whom received treatment with palbociclib and fulvestrant while 26 were treated with everolimus and exemestane as a second-line treatment. All patients had received at least one or two lines of prior endocrine therapy. The median PFS was significantly higher in the patients who received everolimus and exemestane compared with those treated with palbociclib and fulvestrant (6.1 versus 4.5 months; HR: 0.58; 95% CI [0.35–0.96]; *p* = 0.025) [26].

#### 4.1.3. Abemaciclib

Abemaciclib is the third cyclin-dependent kinase 4 and 6 inhibitor (whose molecular formula is C₂₇H₃₂F₂N₈) approved by the FDA for the treatment of advanced or metastatic breast cancer with positive hormone receptors and HER-2 negative.

The MONARCH-2 study was a global phase III, randomised (2:1), double-blind clinical trial which included 669 pre-, peri- (with ovarian suppression), and postmenopausal women with advanced breast cancer, positive hormone receptors, and HER-2 negative, who were resistant to endocrine therapy. One arm received treatment with abemaciclib (150 mg/12 h) and fulvestrant (500 mg), while the other arm received fulvestrant (500 mg) and a placebo. The median survival was 46.7 months for the group receiving abemaciclib and fulvestrant, compared with 37.3 months for the placebo and fulvestrant group (HR: 0.757; 95% CI [0.606–0.945]; *p* = 0.0137). Additionally, this benefit was more pronounced in patients with visceral metastasis (HR: 0.675) and primary resistance to prior endocrine therapy (HR: 0.622). Furthermore, the time to require chemotherapy was also improved for the patients treated with abemaciclib compared with those in the placebo group (HR: 0.622; 95% CI [0.499–0.775]) [27,28].

Following the same line, MONARCH-3 was a phase III, randomised (2:1), double-blind clinical trial which included 493 patients with breast cancer, positive hormone receptors, and HER-2 negative who presented with locoregional recurrence or distant metastasis. The patients were randomised such that one group received abemaciclib (150 mg/12 h) or a placebo, in addition to a non-steroidal aromatase inhibitor (NSAI) (anastrozole (1 mg) or letrozole (2.5 mg)). The majority of the patients (79.1%) received letrozole. The median follow-up time was 17.8 months. The median PFS was 14.7 months in the placebo-treated arm, and up to the cut-off date, the data for the abemaciclib and NSAI treatment arm were not available (HR: 0.54; 95% CI [0.41–0.72]; *p* = 0.00021). Although the data were still immature, the median survival was similar between both arms of the study, with 32 (9.8%) deaths in the abemaciclib arm and 17 (10.3%) deaths in the placebo arm (HR: 0.97) [29].

Additionally, the MONARCH study was more innovative, as it introduced a third treatment arm. Thus, MONARCH was a phase II, multicentric, randomised (1:1:1), open-label clinical trial which included 234 women with metastatic breast cancer, positive hormone receptors, and HER-2 negative who had progressed after receiving endocrine therapy and chemotherapy. The median follow-up was 27.2 months (cut-off date: 28 June 2019). In one treatment arm, patients received 150 mg of abemaciclib and 20 mg of tamoxifen. In another, they received only 150 mg of abemaciclib, and in the third arm, they were given 200 mg of abemaciclib along with prophylactic loperamide (antidiarrhoeal, opioid μ receptor agonist). The median survival was 24.2 months for the group combining abemaciclib and tamoxifen; 20.8 months for the patients who received 150 mg of abemaciclib monotherapy (combination therapy versus 150 mg of abemaciclib (HR: 0.620; 95% CI [0.397–0.969]; *p* = 0.034)), and 17.0 months for those who received 200 mg of abemaciclib with loperamide (150 mg abemaciclib versus 200 mg abemaciclib (HR: 0.956; 95% CI [0.635–1.438]; *p* = 0.832)) [30].

Finally, MONARCH + was a phase III, randomised, double-blind clinical trial which included 463 postmenopausal women from China, Brazil, India, and South Africa who had advanced breast cancer, positive hormone receptors, and HER-2 negative [31].

The participants were divided into two cohorts [31]:

- Cohort A (308 patients) had not received prior systemic treatment. A total of 207 patients were treated with abemaciclib (150 mg/12 h), and 99 patients received a placebo. Additionally, both arms received treatment with anastrozole (1 mg/24 h) or letrozole (2.5 mg/24 h).
- Cohort B (157 patients) had progressed after receiving endocrine therapy. A total of 104 participants received treatment with abemaciclib (same dose as Cohort A), and 53 patients were assigned to the placebo group. Both treatment arms also received fulvestrant (500 mg).

In Cohort A, the median PFS was not reached in the abemaciclib-treated group, while it was 14.7 months in the placebo arm. The 12-month PFS rate was 72.1% for patients in the abemaciclib arm compared with 58.0% for those in the placebo group (*p* = 0.0207). In Cohort B, the median PFS was 11.5 months in the abemaciclib-treated group and 5.6 months in the placebo arm (HR: 0.376; 95% CI [0.240–0.588]; *p* < 0.0001). Additionally, the 12-month PFS rates were 49.1% for the group which received abemaciclib compared with 28.9% for the placebo group (*p* = 0.0229) [31].

#### 4.1.4. Dalpiciclib

Dalpiciclib is another selective cyclin-dependent kinase 4 and 6 inhibitor (whose molecular formula is C₂₄H₂₉N₇O₂), though it has not yet been approved for the treatment of breast cancer. Its use in this pathology was evaluated in the DAWNA-1 study.

DAWNA-1 was a phase III, randomised (1:2), double-blind, placebo-controlled clinical trial which included 361 women with advanced breast cancer, positive hormone receptors, and HER-2 negative whose disease had progressed after receiving two lines of endocrine therapy prior to inclusion in the study. A total of 241 patients received treatment consisting of dalpiciclib and fulvestrant, and 120 patients were given a placebo and fulvestrant. The dalpiciclib group was followed for a median of 10.7 months, while the placebo group had a median follow-up of 10.6 months (cut-off date: 15 November 2020). Regarding the patients’ characteristics, the median age was 51 years, with most (55.7%) being postmenopausal women, while 60.1% had visceral metastasis. In addition, 27% of the patients in the dalpiciclib and fulvestrant arm and 40.8% of those in the placebo group received at least one additional round of antitumour therapy after discontinuing the trial treatment. By the cut-off date, 86 (35.7%) and 76 (63.3%) deaths or disease progressions had occurred in the dalpiciclib and placebo groups, respectively. Moreover, the median PFS was 15.7 months in the dalpiciclib group, compared with 7.2 months in the placebo group (HR: 0.42; 95% CI [0.31–0.58]; *p* < 0.0001). At 6 months, the PFS rate in the dalpiciclib and fulvestrant arm was 76.4% (95% CI [70.1–81.5]), compared with 53.2% in the placebo and fulvestrant group (95% CI [43.5–62.0]). At 12 months, these rates were 51.8% (95% CI [43.2–59.8]) and 29.1% (95% CI [20.2–38.5]), respectively. The median survival was still immature at the time of analysis [32].

#### 4.1.5. Inhibitors of Cyclin-Dependent Kinase 4 and 6 (iCDK4/6)

PRAEGNANT was a study which included 1803 women with breast cancer, positive hormone receptors, and HER-2 negative. The included patients received treatment with an iCDK4/6 in combination with endocrine therapy or endocrine therapy alone and were divided according to whether the treatment corresponded to first-, second-, or third-line therapy. Thus, in the first-line treatment, the median PFS was 24.7 months for those receiving combined iCDK4/6 and endocrine therapy (95% CI [11.9–not reached]) compared with 16.6 months for those treated with endocrine therapy alone (95% CI [10.9–22.6]). In the second-line treatment, the median PFS was 7.8 months (95% CI [5.8–15.4]) for patients receiving combined therapy versus 8.7 months for the monotherapy group (95% CI [6.0–11.5]). Finally, in the third-line treatment, the median PFS was 4.7 months (95% CI [3.4–8.3]) for the group treated with endocrine therapy alone, compared with 4.2 months (95% CI [3.0–14.5]) for the combined treatment group. At the time of the analysis, the median survival was still immature [33].

As a summary, Table 4 presents the efficacy results of the various studies analysed in this article.

### 4.2. Safety

#### 4.2.1. Ribociclib

The most frequently reported adverse effect in the studies which added ribociclib to endocrine therapy was neutropenia, which occurred with nearly the same frequency regardless of the selected hormone therapy.

Anaemia and infections were less common when ribociclib was combined with letrozole compared with other endocrine therapies. However, the ribociclib-letrozole combination was associated with a higher percentage of patients experiencing leukopenia, diarrhoea, asthenia, and nausea at some moment during treatment.

#### 4.2.2. Palbociclib

The most commonly reported adverse reaction when palbociclib was combined with hormone therapy was once again neutropenia. Palbociclib was the drug which led to the highest number of cases of febrile neutropenia, accounting for 7.6% of all neutropenia cases in the RENATA study.

The second-most-common adverse effect was leukopenia.

Furthermore, the combination with AI (letrozole and anastrozole) led to a higher incidence of anemia compared with the combination of palbociclib with fulvestrant.

#### 4.2.3. Abemaciclib

As well as other cyclin-dependent kinase 4 and 6 inhibitors, the most common adverse effect was neutropenia, followed by leukopenia and anemia.

It is worth noting that when abemaciclib was added to endocrine therapy, it was associated with a higher frequency of vomiting, diarrhoea, thrombocytopenia, and increased transaminases compared with other cyclin-dependent kinase 4 and 6 inhibitors.

#### 4.2.4. Dalpiciclib

In the DAWNA-1 study, in which dalpiciclib was combined with fulvestrant, the most frequently reported adverse effect was neutropenia, as well as other cyclin-dependent kinase 4 and 6 inhibitors. This was followed by leukopenia and anemia.

Infections were also an important adverse reaction in these patients, in contrast to most studies involving other cyclin-dependent kinase 4 and 6 inhibitors, where infections were not commonly reported.

#### 4.2.5. iCDK4/6

The PRAEGNANT study did not specify which cyclin-dependent kinase 4 and 6 inhibitor was used, but the data were similar to those of other studies, with neutropenia, leukopenia, diarrhoea, and asthenia being the most frequent adverse effects.

It should be noted that leukopenia was reported less frequently in studies combining iCDK4/6 with tamoxifen compared with other endocrine therapies.

Finally, Table 5 schematically presents the safety results for these compounds.

## 5. Discussion

The longest PFS was achieved when ribociclib was the cyclin-dependent kinase 4 and 6 inhibitor combined with endocrine therapy. These figures were even greater when the treatment was provided as a first-line therapy. In all of the studies analysed, the differences were statistically significant compared with a placebo.

Regarding OS, with the available data (as some studies still had immature results), higher OS rates were also achieved when endocrine therapy was combined with ribociclib.

It is worth noting that the adverse effects reported in trials studying the addition of ribociclib to endocrine therapy were not observed with greater frequency compared with other CDK4/6 inhibitors. Therefore, ribociclib demonstrated higher efficacy with a similar toxicity profile when compared with the other cyclin-dependent kinase 4 and 6 inhibitors. Additionally, it should be considered that adverse events (neutropenia, leukopenia, nausea, diarrhoea, and asthenia) were more frequent when ribociclib was combined with letrozole, except for anemia and infections, which were more common in patients receiving ribociclib and fulvestrant.

On the other hand, abemaciclib showed broader differences in PFS compared with a placebo when combined with hormonal therapy as a first-line treatment (MONARCH-3).

In addition, for abemaciclib, it was observed in MONARCH-2 and MONARCH-3 that the most frequent adverse effect was diarrhoea (reported in more than 80% of patients). However, there was variability in the figures for other adverse effects reported in these studies. These differences may be due to the use of different treatment strategies, as the studies involved different lines of therapy (second line in MONARCH-2 and first line in MONARCH-3). Both used the same endocrine therapy (fulvestrant); however, adverse events were generally more frequent in MONARCH-3.

As for palbociclib, better PFS rates were achieved when it was combined with endocrine therapy as a first-line treatment. It is not possible to determine whether its effectiveness was greater with one type of endocrine therapy over another, as in all of the studies analysed, palbociclib was always combined with letrozole when used as a first-line treatment. The differences in PFS between the groups treated with palbociclib and those who received a placebo appeared to be more pronounced when palbociclib was used in postmenopausal women. However, it is challenging to draw firm conclusions, as although the studies were conducted exclusively on postmenopausal women (e.g., RENATA, PALOMA-1, and PALOMA-2), in those trials involving premenopausal women, postmenopausal women also participated.

Furthermore, when palbociclib is combined with endocrine therapy (fulvestrant) as a third-line treatment, it has been shown to achieve a lower median PFS compared with patients treated with a combination of everolimus and exemestane.

Regarding the OS figures achieved in the studies analysing palbociclib use in this patient profile, it is noteworthy that although better results were observed in the groups combining palbociclib with endocrine therapy compared with a placebo or endocrine therapy alone, none of the studies with OS data (PALOMA-1 and PALOMA-3) detected statistically significant differences.

As for the safety of palbociclib, its figures did not differ significantly from those of other CDK4/6 inhibitors. However, it is worth noting that the combination of palbociclib with fulvestrant more frequently caused thrombocytopenia compared with its combination with other types of hormonal therapies.

Dalpiciclib nearly doubled the PFS compared with the placebo group in the DAWNA-1 study, a difference which was statistically significant. However, nearly 26% of patients treated with dalpiciclib experienced some type of infection during the treatment period, representing one of the highest infection rates reported among all of the analysed studies. It is worth highlighting that this adverse effect was more frequent when any of the CDK4/6 inhibitors were combined with fulvestrant.

Finally, given that the study included trials with populations from diverse geographical regions, the conclusions may not be universally applicable to the entire population, as the specific characteristics of different groups could have influenced the results. Furthermore, several mechanisms have been described which contribute to resistance to CDK4/6 inhibitors in the treatment of breast cancer, which could affect the interpretation of the results of the presented studies. These mechanisms include overexpression of CDK6, loss of the tumour suppressor protein RB, activation of alternative signalling pathways such as the PI3K/AKT pathway, overexpression of growth factors such as EGF and HER2, and alterations in epigenetic regulation affecting gene expression. However, the combination of CDK4/6 inhibitors with targeted therapies, along with the identification of biomarkers, could help predict treatment response, enabling more personalised therapeutic strategies [34,35,36].

In light of the observed variability in adverse event profiles reported in studies evaluating cyclin-dependent kinase 4 and 6 (CDK4/6) inhibitors alongside endocrine therapy, we conducted a detailed analysis to identify contributing factors influencing this variability:

1. Patient Population Characteristics: We categorised the studies based on demographic parameters, including age, comorbidities, and treatment histories. Our analysis revealed that certain populations, such as older patients or those with multiple comorbidities, exhibited a higher incidence of adverse events, possibly due to altered pharmacodynamics and increased sensitivity to treatment.
2. Type of Endocrine Therapy: We examined the specific endocrine therapies used in conjunction with CDK4/6 inhibitors across various studies. The results indicated that combinations with selective aromatase inhibitors had different adverse event profiles compared with combinations with tamoxifen. For instance, neutropenia was more frequently reported in patients receiving ribociclib paired with letrozole compared with those receiving palbociclib with fulvestrant, suggesting differential interactions between agents.
3. Methodological Variability: Disparities in study designs and reporting criteria were noted. Studies with larger sample sizes tended to provide a more consistent account of adverse events, while smaller trials exhibited significant variability in their reported outcomes. The follow-up durations also played a role, as longer studies provided a more comprehensive assessment of late-emerging adverse events.

Through this analysis, we illuminated key factors contributing to the variability in adverse event reports associated with CDK4/6 inhibitors and their combinations with endocrine therapies.

On the other hand, loco-regional treatment (LRT), which may include surgical interventions such as mastectomy or breast-conserving surgery followed by radiotherapy, has been a topic of considerable debate in the management of de novo MBC. While conventional treatment typically focuses on systemic therapies, the role of LRT in influencing prognosis and oncologic outcomes deserves thorough exploration.

Several studies, including the recent retrospective analysis conducted by Tinterri et al. [37], have reported on the variability in outcomes associated with LRT. In this study, which included 61 de novo MBC patients who underwent front-line chemotherapy followed by LRT, the findings suggest that while LRT may improve local disease control, its impact on overall survival (OS), progression-free survival (PFS), and distant progression-free survival (DPFS) rates was less definitive. After a median follow-up of 55 months, the study revealed disease progression in 60.7% of patients and an OS rate of 58.8% at five years, with no significant survival advantage observed across different patient subgroups. Notably, a trend towards better PFS and DPFS rates was observed among triple-positive tumors, yet this was not statistically significant.

While randomised controlled trials (RCTs) provide the gold standard for evaluating the efficacy and safety of cyclin-dependent kinase 4 and 6 (CDK4/6) inhibitors in combination with endocrine therapy, real-world data (RWD) offer complementary insights into their effectiveness and tolerability in broader patient populations. RWD, collected from routine clinical practice, can help bridge the gap between controlled trial conditions and everyday clinical application, particularly by including patients who may be underrepresented in RCTs, such as those with comorbidities or differing adherence patterns. One notable example is the RENATA study, which offers valuable insights into the real-world use of palbociclib in patients with HR+/HER2− advanced breast cancer, complementing the findings from randomised controlled trials (RCTs) such as PALOMA-2 and PALOMA-3. Through a prospective analysis within a Latin American population, the study demonstrated comparable overall response rates and progression-free survival while also revealing a lower incidence of adverse events and a reduced need for treatment interruptions. Additionally, RENATA highlights the heterogeneity of patient profiles in clinical practice, including those with poor performance statuses and visceral metastases, which underscores the applicability and relevance of real-world findings compared with controlled trials which typically involve more homogeneous populations. This emphasises the importance of integrating real-world data to bridge effectiveness, safety, and patient experience in the management of cancer [25].

## 6. Limitations

Despite the promising findings, this study presents certain limitations which should be considered. Firstly, the selection of studies was restricted to articles published in the last five years and available free of charge, which may have excluded relevant research. Additionally, some of the analysed studies still present immature results, particularly in terms of overall survival, preventing definitive conclusions about the long-term impact of CDK4/6 inhibitors. Likewise, population differences were not extensively addressed, potentially limiting the applicability of the results to different ethnic and geographical groups. Finally, most studies compared CDK4/6 inhibitors with placebo or hormone therapy, with less evidence available on direct comparisons between different drugs in this class, making it challenging to determine which offers the best efficacy and safety profile.

## 7. Conclusions

The addition of CDK4 and 6 inhibitors to endocrine therapy in the treatment of advanced or metastatic breast cancer with positive hormone receptors and HER-2 negative has significantly improved PFS, median survival, and chemotherapy-free intervals compared with the use of hormonal treatments alone or in combination with a placebo.

Ribociclib demonstrated greater efficacy, with a safety profile similar to that of other cyclin-dependent kinase 4 and 6 inhibitors.

Based on the analysed data, it is suggested that palbociclib might be more effective when used in postmenopausal women. However, clinical trials involving only premenopausal women treated with palbociclib are needed to confirm this with certainty.

Currently, cyclin-dependent kinase 4 and 6 inhibitors are becoming established as a new standard treatment for this pathology, potentially offering lower toxicity than chemotherapy, as they are selective inhibitors of cyclin-dependent kinases 4 and 6. In all cases, the most frequent adverse effects were haematological, primarily neutropenia and leukopenia.

However, given that in these patients the disease ultimately progresses, requiring chemotherapy, it is necessary to deeply investigate the mechanisms of treatment resistance and, in the process, develop effective therapies to overcome them.

## Figures and Tables

**Table 1:** Molecular classification of breast cancer. ER = estrogen receptors; PR = progesterone receptors; Ki67 = proliferation index (source: Ignacio Zapardiel Gutiérrez, 2016 [6]).

LUMINAL A	LUMINAL B		HER-2	TRIPLE NEGATIVE
	HER-2 Negative	HER-2 Positive		
RE + RP > 20% HER-2–Ki67 <20%	RE + RP ≤ 20% HER-2-Ki67 ≥ 20%	RE + RP ≤ 20% HER-2+ Ki67 ≥ 20%	RE- RP-HER-2+	RE- RP-HER-2−

**Table 2:** Treatment selection for Luminal A, Luminal B, triple-negative, and HER-2 positive tumours and metastatic breast cancer. HT = hormone therapy; QT = chemotherapy (modified from Ignacio Zapardiel Gutiérrez, 2016 [6]).

TUMOUR TYPE	STAGE	TREATMENT
LUMINAL A	Early Stage	Breast-conserving surgery + axillary staging; If conserving surgery is not possible: - <60 years → HT - >60 years → mastectomy + axillary staging
	Locally Advanced	- <60 years → HT - >60 years → mastectomy + axillary staging
LUMINAL B HER2 -	<2–3 cm	Breast-conserving surgery + axillary staging; If conserving surgery is not possible: - <70 years → neoadjuvant QT - >70 years → neoadjuvant HT or QT
LUMINAL B HER2 +	<2 cm	Breast-conserving surgery + axillary staging; If conserving surgery is not possible: - Without comorbidity → neoadjuvant QT + trastuzumab - With comorbidity or advanced age → adapted neoadjuvant QT + trastuzumab or surgery
TRIPLE NEGATIVE	<2 cm	Breast-conserving surgery If conserving surgery is not possible → neoadjuvant QT
HER2+	<2 cm	Breast-conserving surgery + axillary staging If conserving surgery is not possible → neoadjuvant QT + trastuzumab
METASTATIC BREAST CANCER	-	Hormone therapy, chemotherapy, targeted therapy

**Table 3:** PICO strategy.

P	Women with advanced or metastatic breast cancer with positive hormone receptors and HER-2 negative
I	Hormone therapy + iCDK4/6
C	Placebo or hormone therapy, monotherapy or other adjuvant therapies
O	Safety and efficacy of iCDK 4/6 in combination with hormone therapy in patients with advanced or metastatic breast cancer with positive hormone receptors and HER-2 negative

**Table 4:** Efficacy results of the different studies. RIBO = ribociclib; LET = letrozole; PBO = placebo; FUL = fulvestrant; TAM = tamoxifen; NSAI = nonsteroidal aromatase inhibitors; ABE = abemaciclib; PAL = palbociclib; EVE = everolimus; EXE = exemestane; AI = aromatase inhibitor; DAL = dalpiciclib; iCDK4/6 = cyclin-dependent kinase 4 and 6 inhibitors; ET = endocrine therapy; ETmono = endocrine monotherapy; Pts = patients; HR = hazard ratio; CI = confidence interval. The question mark (?) means that the data corresponding to the time of the study was not provided.

DRUG	CLINICAL TRIAL	AUTHORS	COUNTRY OF ORIGIN	CONTEXT	NUMBER OF PATIENTS AND AVERAGE FOLLOW-UP TIME	TYPE OF PATIENTS	EFFECTIVENESS	
							PROGRESSION-FREE SURVIVAL (PFS; Months)	GLOBAL SURVIVAL (OS; Months)
	MONALEESA-2	Hortobagyi GN et al.	USA (multicentric)	1st line	668 pts 79.7 months (June 2021)	Postmenopausal women	RIBO + LET: 25.3 PBO + LET: 16.0 (HR, 0.56; 95% CI, 0.43–0.72; *p* < 0.001)	RIB + LET: 63.9 PBO + LET: 51.4 (HR, 0.76, 95% CI, 0.63–0.93; *p* = 0.04)
							In June 2019 analysis:	
							RIB + FUL: 20.5 (33.6 en 1st line)	
							PBO + FUL: 12.8 (19.2 en 1st line)	RIB + FUL: 53.7
RIBOCICLIB	MONALEESA-3	Slamon DJ et al.	USA (multicentric)	1st and 2nd line	726 pts 56.3 months (October 2020)	Postmenopausal women	(HR, 0.593; 95% CI, 0.480–0.732; *p* < 0.001) In October 2020 analysis: RIB + FUL: 37.4 PBO + FUL: 28.1 (HR, 0.7069, 95% CI 0.57–0.84)	PBO + FUL: 41.5 (HR, 0.724; 95% CI, 0.568–0.924; *p* = 0.00455)
	MONALEESA-7	Finn RS et al.	USA (multicentric)	1st and 2nd line	672 pts 53.5 months (June 2020)	Postmenopausal women	RIBO + TAM/NSAI: 23.8 PBO + TAM/NSAI: 13.0 (HR, 0.55; 95% CI, 0.44–0.69; *p* < 0.0001)	RIBO + TAM/NSAI: 58.7 PBO + TAM/NSAI: 48.0 (HR, 0.76; 95% CI, 0.61–0.96; *p* = 0.00973)
	MONARCH-2	Sledge GW et al.	USA (multicentric)	2nd line	669 pts 47.7 months (February 2017)	Pre and postmenopausal women	ABE + FUL: 16.4 PBO + FUL: 9.3 (HR, 0.553; 95% CI, 0.449–0.681; *p* < 0.001)	ABE + FUL: 46.7 PBO + FUL: 37.3 (HR, 0.757; 95% CI, 0.606–0.945; *p* = 0.0137)
	MONARCH-3	Goetz MP et al.	USA (multicentric)	1st line	493 pts 17.8 months (?)	Postmenopausal women	ABE + NSAI: 28.18 PBO + NSAI: 14.76 (HR, 0.540; 95% CI, 0.418–0.698; *p* = 0.000002)	-
								ABE + TAM: 24.2
								ABE 150 mg: 20.8
	Next MONARCH	Hamilton EP et al.	USA and European centers	2nd line	234 pts 27.2 months (June 2019)			ABE 200 mg: 17.0
ABEMACICLIB						-	-	(ABE + TAM vs. ABE 150 mg: HR 0.620 (95% CI, 0.397–0.969 *p* = 0.034); ABE 150 mg vs. ABE 200 mg: HR 0.956 (95% CI, 0.635–1.438 *p* = 0.832)
							Cohort A:	
							ABE + ET: not achieved	
	MONARCH plus	Zhang QY et al.	China (multicentric)	1st and 2nd line	306 pts in cohort A (1st line) 157 pts in cohort B (2nd line) 16 months	Postmenopausal women	ABE + PBO: 14.7 (HR, 0.499; 95% CI, 0.346–0.719; *p* = 0.0001) Cohort B: ABE + ET: 11.5 PBO + ET: 5.6 (HR, 0.376; 95% CI, 0.240–0.588; *p* < 0.0001)	-
PALBOCICLIB	PALOMA-1	Finn RS et al.	USA (multicentric)	1st line	165 pts 64.7 months (December 2016)	Postmenopausal women	PAL + LET: 20.2 LET: 10.2 (HR, 0.488; 95% CI 0.319–0.748; *p* = 0.0004)	PAL + LET: 37.5 LET: 34.5 (HR, 0.897; 95% CI, 0.623– 1.294; *p* = 0.281)
	PALOMA-2	Finn RS et al.	USA (multicentric)	1st line	267 pts 37.5 months (May 2017)	Postmenopausal women	PAL + LET: 24.8 PBO + LET: 14.5 (HR, 0.58; 95% CI, 0.46–0.72; *p* < 0.001)	Immature
	PALOMA-3	Rugo HS et al.	USA (multicentric)	1st line	521 pts 44.8 months (April 2018)	Pre- and postmenopausal women	PAL + FUL: 9.5 PBO + FUL: 4.6 (HR, 0.46; 95% CI, 0.36–0.59; *p* < 0.0001)	PAL + FUL: 34.9 PBO + FUL: 28.0 (HR, 0.81; 95% CI, 0.64–1.03; *p* = 0.09)
	PALOMA-4	Xu B et al.	China (multicentric)	1st line	340 pts 52.8 months (August 2020)	Postmenopausal women	PAL + LET: 21.5 PBO + LET: 13.9 (HR, 0.68, 95% CI, 0.53–0.87; *p* = 0.0012).	-
	Palbociclib + Fulvestrant or Everolimus + Exemestano	Orlandi et al.	Multicenter	3rd line	74 pts	Pre- and postmenopausal women	PAL + FUL: 4.5 EVE-EXE: 6.1 (HR, 0.58, 95% CI, 0.35–0.96, *p* = 0.025)	-
	RENATA	Petracci F et al	Latin America	1st and 2nd line	128 pts	Pre- and postmenopausal women	PAL + AI/FUL: 1st line = 36.7, 2nd line = 24.2	Not achieved
DALPICICLIB	DAWNA-1	Delord JP et al.	France (multicentric)	2nd line	361 pts 10.7 months (November 2020)	Pre- and postmenopausal women	DAL + FUL: 15.7 (CI not achieved) DAL + PBO: 7.2 (HR, 0.42, 95% CI, 0.31–0.58; *p* < 0.0001)	Not measured
iCDK4/6	PRAEGNANT	Scheneeweiss A et al.	Germany	1st and 2nd line	1803 pts 24 months	-	1st line: iCDK4/6 + ET: 25.7 (CI not achieved possible small sample) ETmono: 16.6 (95% CI, 10.9–22.6) 2nd line: iCDK4/6 + ET: 7.8 (95% CI, 5.8–15.4) ETmono: 8.7 (95% CI, 6.0–11.5) 3rd line: iCDK4/6 + ET: 4.2 (95% CI, 3.0–14.5) ETmono: 4.7 (95% CI, 3.4–8.3)	Not measured

**Table 5:** Safety results of the studies analysed in this study.

DRUG	CLINICAL TRIAL	AUTHORS	COUNTRY OF ORIGIN	ENDOCRINOTHERAPY	% PATIENTS WHO SUFFERED ADVERSE EFFECTS							
					FEBRILE NEUTROPENIA	LEUKOPENIA	ANEMIA	INFECTIONS	NÁUSEA	DIARRHOEA	ASTHENIA	OTHERS
		Finn RS et al.	USA (multicenter)						30			
	PALOMA-1			Letrozol	75/0	43	41	-	(vomiting:	22	41	-
									18)			
	PALOMA-2	Finn RS et al.	USA (multicenter)	Letrozol	66.5	24.8	5.4	0	0.2	1.4	1.8	
		Rugo HS et al.	USA (multicenter)									1
	PALOMA-3			Fulvestrant	70/1	-	4	5	0	0	3	Thrombocytopenia
												(3)
PALBOCICLIB	PALOMA-4	XuB et al.	China (multicenter)	Letrozol	84.5/2.4	36.3	4.8					
	Palbociclib + Fulvestrant or Everolimus + Exemestano	Orlandi A	Multicenter	Fulvestrant	65/0	-	41	-	9	0	35	Thrombocytopenia (24)
		Petracci F et al.	Latin America						77.3 (vomiting 77.3)			Constipation (79.4)
	RENATA			AI and Fulvestrant	82/7.6	76.6	77.3	27.5		77.3	77.3	
		Zhang QY et al.	China (multicenter)						45.1			Abdominal pain
	MONARCH-2			Fulvestrant	46/1.35	28.3	29	11.1	(vomiting:	86.4	39.9	(35.4)
									25.9)			
		Goetz MP et al.	USA (multicenter)									Thrombocytopenia
ABEMACICLIB	MONARCH-3			Fulvestrant	80	76.1	62	39.1	26.8 (vomiting: 15.6)	80	29.3	(44.4) Transaminases (34.6)
												Abdominal pain
	nextMONARCH	Hamilton EP et al.	USA and European centers	Tamoxifeno	49.6	30.8	40.6	-	36.3	61.1	29.9	(27.4)
						Cohort A:	Cohort		Cohort A:	Cohort	Cohort A:	
	MONARCHplus	Zhang QY et al.	China (multicenter)	Letrozol and Anastrozol	Cohort A: 80 Cohort B: 80.8	76.1 Cohort	A: 62 Cohort	-	26.8 Cohort B:	A:80 Cohort	29.3 Cohort B:	Thrombocytopenia (44.4; 41.3)
						B:82.7	B: 70.2		18.3	B:78.8	23.1	
RIBOCICLIB	MONALEESA-2	Hortobagyi GN	USA (multicenter)	Letrozol	59.3	21	1.2	4.2	2.4	1	2.4	-
	MONALEESA-3	Slamon DJ	USA (multicenter)	Fulvestrant	57.1	15.5	3.9	7.7	1.4 (vomiting: 14.1)	0.6	1.7	
	MONALEESA-7	Finn RS	USA (multicenter)	NSAI (Letrozol and Anastrozol) or Tamoxifeno	63.5	-	-	-	-	-	-	Transaminases (11)
DALPICICLIB	DAWNA-1	Delord JO	France (multicenter)	Fulvestrant	84.2/0	62.1	61.3	25.9	18.8	-	14.6	Transaminases (35)
iCDK4/6	PRAEGNANT	Schneeweiss A	Germany	Endocrinotherapy	11.3	11.3	5	0.7	11.3	5.7	11.3	-

## Data Availability

Not applicable.

## Abbreviations

The following abbreviations are used in this manuscript:

iCDK4/6	Cyclin-dependent kinase 4 and 6
HER-2	Human epidermal growth factor receptor 2
ER	Estrogen receptor
PR	Progesterone receptor
Ki67	Proliferation index
RB	Retinoblastoma protein
E2F	Transcription factor for DNA replication
CDK	Cyclin-dependent kinase
HR	Hazard ratio
NSAI	Non-steroidal aromatase inhibitor
AI	Aromatase inhibitor
CI	Confidence interval
OS	Overall survival
PFS	Progression-free survival
